# Reply to Pérez-García et al.: Perfect is the enemy of good

**DOI:** 10.1073/pnas.2206500119

**Published:** 2022-07-25

**Authors:** Sebastian Dunnett, Robert A. Holland, Gail Taylor, Felix Eigenbrod

**Affiliations:** ^a^Geography and Environmental Science, Faculty of Environmental and Life Sciences, University of Southampton, Southampton SO17 1BJ, United Kingdom;; ^b^Biological Sciences, Faculty of Environmental and Life Sciences, University of Southampton, Southampton SO17 1BJ, United Kingdom;; ^c^Plant Sciences, University of California, Davis, CA 95616

We thank Pérez-García et al. (1) for their letter in response to our recent article (2) reiterating the significant impact of wind turbines on volant species.

First, a point of clarification is needed. Renewable energies are not merely “effective”: they are absolutely critical. The Intergovernmental Panel on Climate Change warns that warming cannot be limited to 2 °C or 1.5 °C without "rapid and deep and in most cases immediate GHG [greenhouse gas] emissions reductions" (3, p. 28). In scenarios limiting global warming to 2 °C, low-carbon sources produce 93 to 97% of global electricity by 2050; it is in this vital context that our study was conducted.

Vultures themselves are not immune from the devastating effects of an unmitigated climate; it is suspected that climate change may adversely affect populations of Bearded and Cape vultures, something flagged as needing more research (4). Indeed, of the 11 endangered species of vulture (1), 3 have climate change listed as a threat alongside renewable energy (5).

We entirely agree that overlap with important conservation areas (ICAs) is not very informative; this part of our analysis aimed to place our results in the context of recent studies. If the recent analysis of Wauchope et al. (6) is anything to go by, the fate of global biodiversity rests solely outside of protected areas. However, the “minimal overlap” derives from a priority ranking calculated using three facets of conservation: species richness, ecoregions, and threat (7); the vulture prioritization layers presented by Pérez-García et al. (1) use two of these (8).

**Fig. 1.**
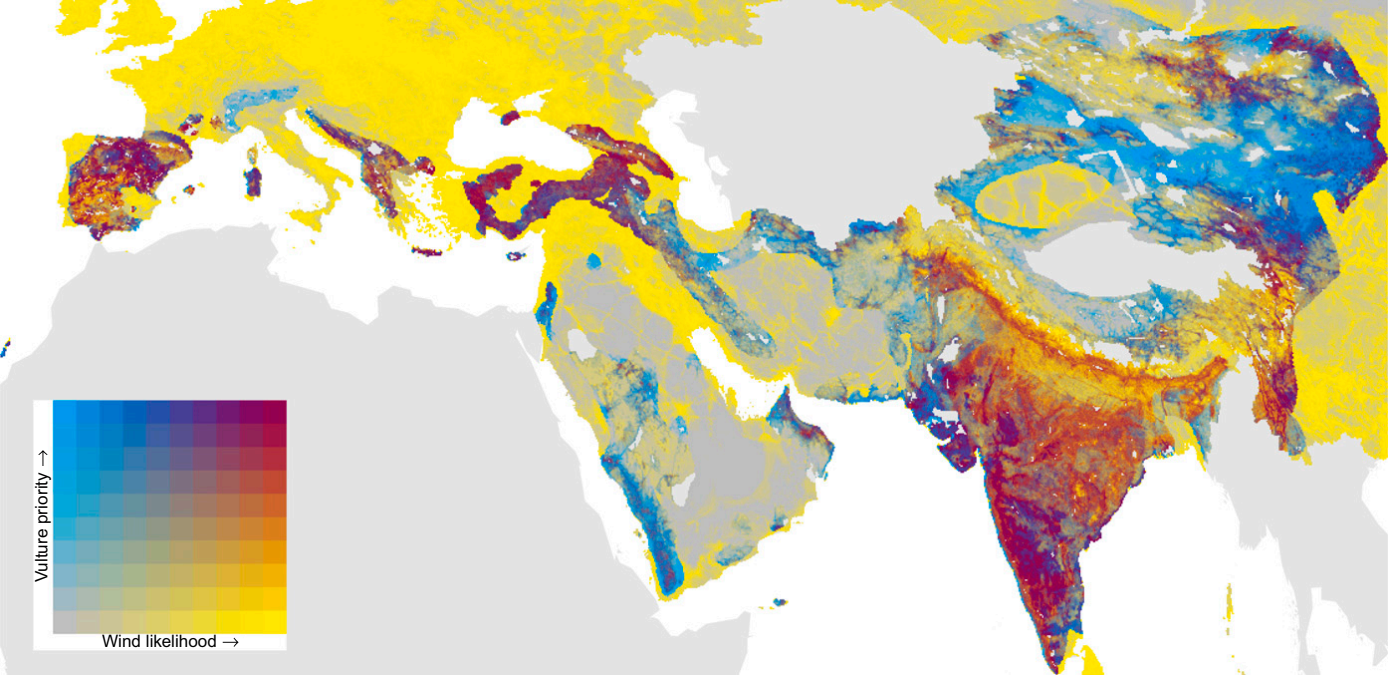
Overlap between priority areas for Old World vulture conservation (PAVC) and wind expansion likelihood. Magenta cells represent the highest risk of impacts with wind farms. Wind likelihood is the predicted probability (zero to one) that an energy installation is present in a given grid cell (taken from ref. 2). PAVC ranks global cells from low to high priority (zero to one) according to the breeding and resident range of the 15 Eurasian vulture species (taken from ref. 2). PAVC rankings outside breeding and resident ranges are assumed to be zero.

In our paper, we suggest that our analysis “allows the direct local impacts of renewable energy … to be interrogated and potentially mitigated” and that “minimal overlap” requires “appropriate policy and regulatory controls” (2). Pérez-García et al. (1) provide a perfect example using data from Thaxter et al. (9). By our estimate, Europe has almost three times as many turbines per area than North and South America combined (10). France has 25 times more per area than the United States, and Germany and Spain 10 times; however, collision figures show many more in the United States than in any European country (Fig. 1) (2). This strongly suggests that something other than pure turbine numbers drives collisions.

Wind turbines can undoubtedly have a significant impact on soaring birds. However, our analysis and the collision data open up the possibility of high turbine densities existing with low collision rates; we welcome, and indeed strongly encourage, any further research to determine in what context this occurs. Finally, often avoidance of known impacts will be straightforward. Fig. 1 adapts Figure 2 in Pérez-García et al. (1) to include wind resource immediately outside of vulture ranges.
